# Patient adherence as a predictor of acute and long-term outcomes in concentrated exposure treatment for difficult-to-treat obsessive-compulsive disorder

**DOI:** 10.1186/s12888-024-05780-6

**Published:** 2024-04-30

**Authors:** Kristian Tjelle, Håvard Berg Opstad, Stian Solem, Gerd Kvale, Michael G. Wheaton, Thröstur Björgvinsson, Bjarne Hansen, Kristen Hagen

**Affiliations:** 1 Department of Psychiatry, Møre og Romsdal Hospital Trust, Molde Hospital, Molde, 6412 Norway; 2 Department of Psychology, Norwegian University of Science and Technology, Trondheim, Norway; 3 Bergen Center for Brain Plasticity, Haukeland University Hospital, Bergen, Norway; 4 Department of Clinical Psychology, University of Bergen, Bergen, Norway; 5 Department of Psychology, Barnard College, New York, NY 10027 USA; 6 Behavioral Health Partial Hospital Program, McLean Hospital, Harvard Medical School, Belmont, MA USA; 7 Department of Psychosocial Sciences, University of Bergen, Bergen, Norway; 8 Department of Mental Health, Norwegian University of Science and Technology, Trondheim, Norway

**Keywords:** OCD, Adherence, ERP, cET, Group, Difficult-to-treat, B4DT, Work- and social functioning

## Abstract

**Background:**

Exposure and response prevention (ERP) is considered the first-line psychotherapy for obsessive-compulsive disorder (OCD). Substantial research supports the effectiveness of ERP, yet a notable portion of patients do not fully respond while others experience relapse. Understanding poor outcomes such as these necessitates further research. This study investigated the role of patient adherence to ERP tasks in concentrated exposure treatment (cET) in a sample who had previously not responded to treatment or relapsed.

**Method:**

The present study included 163 adults with difficult-to-treat OCD. All patients received cET delivered during four consecutive days. Patients’ treatment adherence was assessed using the Patient EX/RP Adherence Scale (PEAS-P) after the second and third day of treatment. OCD severity was evaluated at post-treatment, 3-month follow-up, and 1-year follow-up by independent evaluators.

**Results:**

PEAS-P scores during concentrated treatment were associated with OCD-severity at post-treatment, 3-month follow-up, and 1-year follow-up. Moreover, PEAS-P scores predicted 12-month OCD severity adjusting for relevant covariates. Adherence also predicted work- and social functioning at 1-year follow-up.

**Conclusions:**

These results indicate that ERP adherence during the brief period of cET robustly relates to improvement in OCD symptoms and functioning in both the short and long term. Assessing adherence might identify patients at risk of poor outcomes, while improving adherence may enhance ERP for treatment resistant patients.

**Trial Registration:**

ClinicalTrials.gov identifier: NCT02656342.

## Introduction

Obsessive–compulsive disorder (OCD) is characterized by anxiety-evoking intrusive thoughts, images or urges (obsessions) and repetitive behaviors performed in a ritualistic manner aimed at reducing discomfort (compulsions) [1]. OCD has an estimated lifetime prevalence of approximately 2% [2, 3]. When left untreated, OCD tends to have a chronic course, with low rates of spontaneous remission [4, 5], causing significant distress, functional impairment, affecting interpersonal relations, work status, and reduced quality of life [6–9].

Empirically-supported OCD treatments include cognitive-behavioral therapy (CBT) consisting of exposure and response prevent (ERP) as well as serotonin reuptake inhibitor (SRI) pharmacotherapy, which are both recommended in treatment guidelines as first-line treatments for the disorder [10]. However, approximately 35–40% of OCD-patients respond poorly to these treatments [11–13]. Regarding ERP, the drop-out rate ranges between 25 and 30% in most studies [13], and while the majority of patients (typically 75–80%) experience a treatment response (25–35% decrease in symptoms), usually fewer than half achieve remission (minimal symptoms after treatment [Yale-Brown Obsessive Compulsive Scale scores below 16, 12, or 7]) [14]. Furthermore, although ERP outperforms control conditions, up to 50% of the patients completing traditional ERP do not achieve reliable change [15] at long-term follow-up [16, 17]. ERP non-responders and those who relapse have sometimes been referred to as “treatment resistant” or “difficult-to-treat”. Identifying successful treatments to help this category of OCD sufferers is critically important and has included additional interventions including atypical antipsychotic medications and invasive psychosurgical interventions [10]. Alternatively, some data suggest that additional ERP may be sufficient to help such patients, as additional sessions of ERP can help patients who have not remitted with an initial treatment course to do so [18], while some data suggest that patients who experience a symptom relapse after successful ERP can be helped by an additional exposure exercises [19]. In both of these cases an attractive option would be to deliver ERP in a concentrated form, as this would allow the patient and clinician to know quickly whether a more invasive treatment may be warranted.

Different formats of brief, concentrated, or intensive formats have been developed for OCD. Intensive or concentrated treatments usually entail treatment lasting for less than four weeks. Intensive treatments often contain daily sessions [20]. Previous research has found that 15 sessions of ERP for OCD was equally effective if it was delivered daily for 3 weeks or twice-weekly for 8 weeks and a series of case-studies with matched comparisons groups indicate that CBT for OCD could be equally effective if it was delivered intensively for one week or weekly for 12 to 18 weeks [21, 22]. Storch, Merlo [23] also found that intensive (daily sessions for 3 weeks) was as effective in treating OCD as weekly CBT administered over several weeks. The Bergen 4-day treatment is a concentrated ERP format where the treatment is delivered for four consecutive days and has been described as individual treatment delivered in a group setting. The treatment has previously demonstrated promising results [24–28], and studies investigating the long-term outcome have reported recovery rates of about 70% after 1- year and even at 4-year follow-up [29, 30].

Recent data speak to the success of concentrated ERP (hereafter denoted as cET) as a treatment option for difficult-to-treat OCD. Kvale et al. [31] conducted a large clinical trial that recruited adult OCD patients who had a documented previous trial of ERP and had either not responded or experienced benefit followed by subsequent symptom relapse. Participants were randomized to receive cET with the addition of D-cycloserine (DCS), a partial N-methyl-D-aspartate receptor agonist that has been investigated for its potential to facilitate extinction learning or pill placebo. Although the results showed that DCS failed to potentiate the effects of ERP relative to placebo, the overall outcomes for the sample highlight the benefits for cET for this population: 83.9% of the sample experienced a treatment response (35% reduction in symptoms) while 56.5% achieved remission (35% reduction and a Yale-Brown Obsessive Compulsive Scale score of 12 points or lower at 1-year follow-up). Despite participants being categorized as difficult-to-treat, these response and remission rates compare favorably to the general efficacy of ERP as reported in meta-analyses [12]. Importantly however, the variability in response leaves room for improvement, and necessitates research to identify which patients are less likely to benefit from this treatment and which factors promote treatment success.

Research on predictors of ERP outcomes has sought to answer these important questions. Although many candidate predictors have been identified, the existing literature is characterized by many mixed findings [32]. Potential moderators of treatment outcome include pre-treatment severity of OCD and anxiety, past CBT treatment, avoidance, unemployment, and being single, as well as during-treatment variables such as working alliance and treatment adherence [32, 33]. The degree to which patients adhere to ERP treatment elements seems to be a robust predictor of ERP outcomes. Simpson et al. [34] developed a rating scale to quantify the degree to which patients adhere to therapist instructions to complete exposure homework and between-session response prevention and found that patient adherence significantly predicted OCD severity post-treatment in a sample of 30 OCD patients who received twice-weekly sessions of ERP. Moreover, patient adherence during acute ERP also predicted outcome at 6-month follow-up for the same sample [35]. In a subsequent study with an independent sample, Wheaton and colleagues [36] explored the relationship between patient adherence and response to ERP in patients who received ERP (17 sessions) as part of a study investigating augmentation strategies for incomplete response to serotonin reuptake inhibitors. They also found that therapist rated patient adherence predicted OCD-symptom severity post-treatment. In a subsequent study Ojalehto, Abramowitz [37] found that patient adherence to ERP homework predicted improvement in OCD-symptoms at post-treatment but not at follow-up in a sample of 50 OCD patients receiving twice-weekly ERP with added components of Acceptance and Commitment Therapy (ACT) for OCD.

Adherence to ERP procedures also appears to matter during concentrated ERP. Tjelle et al. [38] reported that adherence was significantly correlated with Y-BOCS scores at post-treatment and 3-month follow-up while controlling for age, sex, and pre-treatment scores in a sample of 42 patients treated with cET. Specifically, patients with greater adherence showed less severe OCD symptoms, higher functioning, and greater well-being at follow-up. None of the patients in this sample however, had received ERP treatment prior to this study (i.e., an ERP naïve sample). This begs the question of whether the same relationship could also be applicable to OCD-patients labelled as difficult-to-treat. This remains an important unstudied question, as there could be differences underlying therapeutic change in difficult-to-treat patients who already tried ERP as compared to ERP naïve patients [31]. Moreover, this past study only evaluated follow-up outcomes at 3-months post-treatment, and therefore it remains an unanswered question if adherence during cET relates to improvement in the longer term.

The aim of the present study was therefore to test whether patient adherence to ERP during concentrated delivery predicts outcomes at both short- and long-term follow-up in a sample of difficult-to-treat OCD patients. We explored whether (1) patient adherence would be negatively correlated with OCD-severity at post-treatment, 3-month and 1-year follow-up; (2) whether PEAS-P would also relate to work- and social functioning at 1-year follow-up; and (3) whether patient adherence would predict OCD severity, work- and social functioning at 1-year follow-up after controlling for other relevant predictors.

## Methods

### Participants and procedure

The present study involved secondary data analysis of the previously published trial that that demonstrated that D-Cycloserine (DCS) did not improve treatment cET outcomes for difficult-to-treat OCD patients [31]. The patent study used a triple blind, three-armed, placebo-controlled design in which patients within each stratum were randomized to 100 mg D-Cycloserine, 250 mg D-Cycloserine, or placebo in a 2:2:1 ratio. Since there were no significant differences between the three conditions, the conditions have in this paper been merged.

Included patients (*N* = 163) had to meet criteria for OCD according to the Diagnostic and Statistical Manual of Mental Disorders-Fifth Edition (DSM-5; 1), able to receive outpatient treatment, at least 18 years old, and fluent in Norwegian. They were enrolled based on being identified as difficult-to-treat based on having either having responded and relapsed, or not responded to previous ERP treatment. The treatment received was either intensive 4-day ERP [29], 3-week inpatient ERP [39], group-based ERP [40], videoconference-based ERP [41], or standard 16-session outpatient ERP [42]. At least six completed sessions of previous ERP had to be documented. Response was defined by ≥ 35% reduction and a post-treatment Y-BOCS score of ≤ 15. Relapse was defined by responding to treatment followed by a ≥ 35% increase in Y-BOCS score from post-treatment to follow-up, a Y-BOCS score of 16 or more. Non-responders were defined as those with a reduction in Y-BOCS scores from pre- to post-of less than 35%, and a Y-BOCS score of ≥ 16 after treatment. Finally, there had to be a minimum of four weeks since the original treatment ended.

Patients with ongoing substance abuse/dependence, bipolar disorder or psychosis, suicidal ideation or plans, intellectual disability (based on previous medical history), and living > 1-hour drive by car/train from the treatment location were excluded. Also, patients were excluded if antidepressants had not been stabilized for at least 12 weeks or participants were unwilling to remain on stable dosage during the four intervention days. Participants who were unwilling to refrain from anxiety reducing substances, such as anxiolytics (e.g., benzodiazepines) and alcohol during the two days of exposure, were excluded. Additional exclusion criteria related to the study included pregnancy or breast feeding, renal impairment, hypersensitivity to D-Cycloserine, porphyria, and epilepsy (see [31]. Patients were assessed at post-treatment, as well as at 3-month and 1-year follow-up.

The sample had a mean age of 34.59 (SD = 10.9) years of age and the majority were female (71.8%). The average duration of OCD was 16.2 (SD = 10.2) years. Baseline symptom severity was moderate to severe symptoms of OCD for the sample overall. In addition, the sample showed moderate symptoms of depression and generalized anxiety. A total of 38.7% did not respond to their last treatment for OCD while 61.3% had relapsed following their last treatment. Almost half of patients (44.7%) received some type of disability benefit indicating affected work ability, while 34.8% worked, and 20.5% were students. A total of 76 patients used some type of psychotropic medication, of which 52 of these (31.9%) used SSRIs. These were required to be at a stable dose prior to enrollment in the present study. The sample’s baseline characteristics are summarized in Table 1.

**Table 1:** Pre-treatment patient characteristics

Characteristic	M (SD)/%
Age	34.6 (10.9)
Percent female	71.8
Duration of OCD (years)	16.2 (10.2)
Duration last treatment (hours)	26.6 (11.0)
Any comorbid disorder	69.3
Number of comorbid disorders	1.7 (1.9)
Reported OCD in the family	39.3
Years in school	11.9 (3.9)
Pre-treatment Y-BOCS score	27.0 (3.9)
Employment	
Work	34.7
Student	20.4
Disability	44.9
Previous treatment outcome	
Non-responder	61.3
Relapse	38.7
Using any psychotropic medication	46.6
Using SSRI	31.9

### Treatment

The Bergen 4-Day Treatment (B4DT) is a concentrated exposure treatment format for OCD, where the treatment is delivered over four consecutive days [29]. Treatment is delivered in groups of 3–6 patients, with a therapist-to-patient ratio of 1:1, ensuring individualized therapeutic interactions. The first day consists of a 3-hour session dedicated to psychoeducation. The focus is on establishing an understanding of OCD and the treatment format. The following two days focus on exposure tasks, conducted in various environments relevant to the individual. Treatment specifically targets the intention behind the exposures and how to avoid avoidance behaviors, adopting a strategy referred to as “leaning into the anxiety (LET)”. On the third day, a 1-hour psychoeducational seminar is provided for the patients’ relatives and friends, aiming to help them understand the treatment process and how they can provide support. The treatment concludes on the fourth day with a comprehensive review of their acquired experiential knowledge, underscoring the importance of continued use of the treatment principles.

### Measures

*The Patient EX/RP Adherence Scale- Patient (PEAS-P)* is a self-report questionnaire consisting of three different items. These assess the patient’s adherence to the therapist’s ERP instructions between sessions. The PEAS was originally developed as a clinician-rated instrument, but in our study it was rated by patients. The PEAS was not adjusted in any way for the patients. A previous study [38] has reported on both clinician- and patient-rated versions of the PEAS. They found that patients rated their adherence slightly lower than therapists (5.7 vs. 6.2).

In this study, PEAS-P was rated by the patient the end of day 2 and 3. The three items are rated on a scale from 1 to 7 where higher scores indicate higher adherence. The first item measures the amount of assigned exposures that were attempted, the second item measures quality of attempted exposures, and the third item measures the degree of success with response prevention. The scale demonstrates good face and content validity as well as excellent interrater reliability [43]. Adherence was rated by the patients either at the evening/night or at the morning before the treatment started the following day, and included both the therapist assisted exposures and the patients’ homework in the afternoon. The PEAS has good content validity and excellent inter-rater reliability [43]. The PEAS-P scores are summarized in Table 2.

*The Yale-Brown Obsessive-Compulsive Scale* (Y-BOCS; [44]) is recognized as the gold standard for assessing severity of OCD symptoms [13]. The scale is completed by interview with an experienced clinician and includes 10 different items, rated on a 5-point scale ranging from 0 (no symptoms) to 4 (severe symptoms). This yields a total score of OCD symptom severity that ranges from 0 to 40 and contains sub scores for obsessions (range 0 to 20) and compulsions (range 0 to 20) [45]. Y-BOCS was rated using blinded assessors.

*The Work and Social Adjustment Scale* (WSAS; [46]) is a questionnaire containing five items. These focus on an individual’s impairment in areas of work, social and private activities, functioning at home and close relationships. The items are individually rated on a 9-point scale ranging from 0 (not at all) to 8 (very severely). The times of measurements were made pre-treatment and at 3-month and 1-year follow up. Total scores thus range from 0 to 40, with higher scores indicating higher levels of functioning impairment. The WSAS demonstrates good internal consistency and test–retest reliability [47].

*Measures of anxiety and depression*. As control variables to test the robustness of the effects of PEAS-P as a predictor of outcomes, we included baseline measures of anxiety and depressive symptomatology. Specifically, patients completed self-report scales including the Generalized Anxiety Disorder-7 (GAD-7; [48]) and Patient Health Questionnaire-9 (PHQ-9; [49]), two commonly utilized research measures for quantifying symptoms of generalized anxiety and depression respectively which both have excellent psychometric properties.

### Statistical analyses

To investigate the relationship between adherence and OCD symptoms pre, post, and follow-up, we used Pearson’s correlations analysis. We also conducted two regression analyses to examine the relationship between adherence and OCD symptoms (Y-BOCS) and functioning (WSAS) at 1-year follow-up, controlling for pre-treatment severity of the dependent variable, sex, age, and severity of anxiety (GAD-7) and depression (PHQ-9). VIF values suggested no problems with multicollinearity in the regression analyses. Missing data was present for 11.1% of the values and was missing completely at random (Little’s MCAR test = 106.6, *p* = .70). Missing values were not replaced, leaving a total of 125 participants with complete data for the regression analysis using Y-BOCS, and 85 when WSAS was the dependent variable. Squared semi-partial correlations were included in the regression analyses which expresses increment in R-square when adding an independent variable. These values indicate variable importance and allows ranking of variables from highest to lowest.

## Results

A correlation analysis showed that patient adherence for both the first and the second day of cET treatment were negatively correlated with OCD-severity (Y-BOCS) at post-treatment, and at 3-month and 1-year follow-up. There were, however, no significant correlation between Y-BOCS pre-treatment and treatment adherence (see Table 3). Patient adherence was negatively correlated with work and social adjustment at 1-year follow-up.

**Table 2:** Item and total scores on PEAS-P at day2, day3

Measure		Min	Max	Mean	SD
PEAS-P Day 2	Total	9	21	17.56	2.34
	Item 1	2	7	6.16	1.06
	Item 2	1	7	5.61	0.99
	Item 3	1	7	5.73	1.21
PEAS-P Day 3	Total	10	21	18.06	2.25
	Item 1	4	7	6.36	0.81
	Item 2	2	7	5.91	0.91
Total	Item 3	1	7	5.79	1.38
PEAS-P Both days		22	42	35.62	4.08

*Note* PEAS-P = Patient EX/RP Adherence Scale

**Table 3:** The correlation between treatment adherence (PEAS-P) and OCD-severity (Y-BOCS)

Measure	PEAS-*P* day 2	PEAS-*P* day 3	PEAS-*P* day 2 + 3
Y-BOCS Pre	.049	.013	.05
Y-BOCS Post	− 0.38***	− 0.37***	− 0.42***
Y-BOCS 3-month F-U	− 0.35***	− 0.34***	− 0.39***
Y-BOCS 1-year F-U	− 0.24**	− 0.20*	− 0.26**
WSAS pre	− 0.01	0.02	0.02
WSAS 1-year F-U	− 0.29**	− 0.31**	− 0.34***

*Note* Y-BOCS = Yale-Brown Obsessive Compulsive Scale, PEAS-P = Patient EX/RP Adherence Scale, WSAS = Work and Social Adjustment Scale, * *p <* .05, ** *p* < .01, *** *p* < .001

A simultaneous multiple linear regression was calculated to predict OCD-severity at 1-year follow-up. This model included demographics (participant age and sex), baseline OCD severity, as well as baseline depression (PHQ-9) and anxiety (GAD-7) severity, and PEAS-P scores. The overall model accounted for 13% of the variance in OCD scores at 12-month follow-up and was significant (*R*^2^ = 0.13, *p* < .001). As shown in Table 4, the PEAS-P emerged as a significant predictor (*β* = − 0.27, *t* = -2.981, *p* = .003) while none of the other variables was significant in the model. The squared semi-partial correlation, a measure of the unique variance explained by each predictor that is equivalent to the *R*^2^ change in a hierarchical model when each predictor is entered previously, indicated that the PEAS-P uniquely accounted for 7% of variance in 12-month YBOCS scores, above and beyond the other variables in the model.

**Table 4:** Regression analysis with Y-BOCS and WSAS as outcome measures

Predictor	β	B	t	*p*	sr^2^
12-month follow-up Y-BOCS					
Baseline Y-BOCS	0.07	0.14	0.79	0.433	0.005
Participant sex	− 0.01	-0.07	-0.05	0.960	< 0.001
Participant age	− 0.10	-0.06	-1.08	0.281	< 0.001
Baseline GAD-7	0.18	0.31	1.69	0.094	0.023
Baseline PHQ-9	0.13	0.17	1.26	0.210	0.013
PEAS-P	− 0.27	-0.49	-2.98	0.003	0.070
12-month follow-up WSAS					
Baseline WSAS	0.43	0.49	4.11	< 0.001	0.179
Participant age	0.08	0.08	0.86	0.393	0.009
Participant sex	− 0.10	-2.19	-0.97	0.331	0.012
Baseline GAD-7	0.20	0.48	1.82	0.073	< 0.001
Baseline PHQ-9	− 0.01	-0.02	-0.12	0.903	0.041
PEAS-P	− 0.35	-0.87	-2.98	< 0.001	0.153

*Note* Y-BOCS = Yale-Brown Obsessive Compulsive Scale; PEAS-P = Patient EX/RP Adherence Scale; WSAS = Warwick-Edinburgh Mental Wellbeing Scale; GAD-7 = Generalized Anxiety Disorder-7; PHQ-9 = Patient Health Questionnaire-9; sr^2^ = squared semi-partial correlation, a measure of the unique variance explained by each predictor that is equivalent to the *R*^2^ change in a hierarchical model when each predictor is entered previously

A similar multiple linear regression was calculated to predict work- and social adjustment (WSAS) at 1-year follow-up. The overall model accounted for 37% of the variance in functioning scores at 12-month follow up and was significant (*R*^2^ = 0.37, *p* < .001). As shown in the table, there was no significant influence of age, sex, depressive symptoms (PHQ-9) and anxiety (GAD-7) but baseline levels of functioning (*β* = 0.43, *t* = 4.11, *p* < .001) and PEAS-P scores (*β* = − 0.35, *t* = -3.76, *p* < .001) both emerged as significant predictors. The squared semi-partial correlations indicated that baseline functioning uniquely accounted for 18% of variance in functioning at 12-month follow-up, with the PEAS-P accounting for 15% of additional outcome variance (see Table 4).

## Discussion

We investigated the relationship between patient adherence to ERP tasks with treatment outcomes in concentrated ERP treatment format for patients identified as difficult-to-treat with ERP. As predicted, we found that there was a significant correlation between self-reported patient treatment adherence and both work and social function and symptom severity post-treatment and at 3-month follow-up and 1-year follow-up.

The results are in line with results from ERP for OCD given in weekly or twice weekly format (e.g., 34, 36), and indicate that patient adherence is just as relevant in concentrated ERP formats as in typical outpatient delivery formats. This study, however, is the first of its kind to look at the role of patient adherence in a difficult-to-treat OCD-sample. The patients enrolled in the present study had all experienced a trial of ERP in the past and had either failed to respond or demonstrated a symptom relapse after achieving improvement. The parent trial showed that cET allowed a substantial portion of these patients to benefit. The present results highlight the importance of adherence to ERP procedures as a predictive marker of treatment success. The sample had relatively high scores on the PEAS-P on both days of the treatment, indicating a relatively high degree of patient adherence on average, which seems to indicate that most patients were able to adhere to the treatment tasks, even though they have a history of either non-response or relapse from previous exposure based treatment. We speculate that the unique format of cET (which includes a combination of group treatment elements and intensive therapist supervised exposures) with intensive 8-hour daily sessions, might have supported the relatively high adherence scores.

A notable new contribution of this analysis is our finding that adherence to ERP tasks during this brief window of treatment robustly predicted outcomes not only at the end of treatment, but also up to a year later. Including covariates in the regression model showed that PEAS-P remained a significant predictor of OCD-severity at 1-year follow-up after controlling for Y-BOCS pre-treatment, sex, age, and baseline scores of depression and anxiety. This suggests that adherence to therapy tasks matters more than these baseline characteristics. This is in line with previous studies that have revealed similar patterns in smaller samples of OCD patients treated with weekly or twice-weekly delivered ERP [34–36]. This strengthens the evidence of patient adherence as a predictor of OCD-severity after exposure-based treatment, and implies that patient adherence is as relevant for concentrated exposure based treatment and for difficult-to-treat OCD-patients. In addition, this is the first study to explore the long-term effect of patient adherence (1-year), and finds that the patient adherence during concentrated exposure treatment predicts long-term OCD symptom outcomes. These findings suggest that patient adherence is an important factor with lasting effects.

Furthermore, the results showed that patients’ ratings on PEAS-P were significantly correlated to work functioning and social adjustment at 1-year follow-up. This is an extension from our previous study, where we found that PEAS-P predicted work and social functioning at 3-month follow-up [38]. Present results show that this predictive relationship remained at longer term follow up of 1-year post-treatment. Interestingly, PEAS-P explains more of the variance in work and social adjustment than in OCD-severity. The finding will thus prove useful as a basis for further research and focus on clinical improvement, as work functioning and social adjustment is widely recognized as a predictor of better quality of life, but often impaired in people suffering from OCD [8]. This also suggest that it may be useful to add broader measurements than symptom severity alone, when examining the role of patient adherence in ERP.

The findings of this study and previous research suggest that adherence is important across different ERP formats and OCD samples. These findings are important as they suggest that it may be possible predict treatment response. Therefore, it could be important to measure adherence throughout treatment. Research also suggests that factors such as therapeutic alliance, treatment expectancy and readiness, avoidance, and insight could be related to adherence [50]. The relation between these variables should therefore be further explored to increase understanding of treatment predictors.

Several study limitations should be acknowledged. One limitation of the study is that it only includes self-report data on adherence. In addition, although PEAS-P was found to be a significant predictor for OCD-severity at 1-year follow-up, it explained a relatively small amount of the variance, indicating that there are other important factors in determining long-term outcomes. The PEAS-P was measured only at the end of the two full days of exposure (8 h), because those days were the ones with the most exposure practices, and we did not collect data on the degree to which patients adhered to ERP principles after the four days of concentrated exposure treatment. This study used data from a pre-registered randomized controlled trial exploring potential potentiating effects of D-Cycloserine, but the secondary analyses included in this paper were not pre-registered. Also, this paper only analyzed a selection of predictors, and their relative effect compared to other potential predictors is unknown. Lastly, the treatment in this study was delivered in a concentrated format, and further studies should therefore try to replicate the findings in other exposure-based treatment formats.

In conclusion, the results of the present study indicate that adherence to the ERP treatment is an important factor in determining both the short and long term outcomes of both OCD severity and work and social adjustment after concentrated exposure treatment (cET) for OCD in difficult-to-treat patients. There is however need for more research regarding strategies to improve patient adherence and thereby potentially improve treatment outcome in difficult to treat patients.

## Data Availability

The dataset for the study is available from the first author upon reasonable request.
